# Pelvic Pain Alters Functional Connectivity Between Anterior Cingulate Cortex and Hippocampus in Both Humans and a Rat Model

**DOI:** 10.3389/fnsys.2021.642349

**Published:** 2021-06-03

**Authors:** Wenjun Yu, Xiaoyan Wu, Yunan Chen, Zhiying Liang, Jinxiang Jiang, Afzal Misrani, Yun Su, Yigang Peng, Jian Chen, Binliang Tang, Mengyao Sun, Cheng Long, Jun Shen, Li Yang

**Affiliations:** ^1^Precise Genome Engineering Center, School of Life Sciences, Guangzhou University, Guangzhou, China; ^2^Department of Radiology, Sun Yat-sen Memorial Hospital, Sun Yat-sen University, Guangzhou, China; ^3^College of Education, Jinggangshan University, Ji’an, China; ^4^School of Psychology, South China Normal University, Guangzhou, China; ^5^School of Life Sciences, South China Normal University, Guangzhou, China

**Keywords:** pelvic pain, anterior cingulate cortex, hippocampus, neural circuits, functional magnetic resonance imaging, electrophysiology

## Abstract

The anterior cingulate cortex (ACC) and hippocampus (HIPP) are two key brain regions associated with pain and pain-related affective processing. However, whether and how pelvic pain alters the neural activity and connectivity of the ACC and HIPP under baseline and during social pain, and the underlying cellular and molecular mechanisms, remain unclear. Using functional magnetic resonance imaging (fMRI) combined with electrophysiology and biochemistry, we show that pelvic pain, particularly, primary dysmenorrhea (PDM), causes an increase in the functional connectivity between ACC and HIPP in resting-state fMRI, and a smaller reduction in connectivity during social exclusion in PDM females with periovulatory phase. Similarly, model rats demonstrate significantly increased ACC-HIPP synchronization in the gamma band, associating with reduced modulation by ACC-theta on HIPP-gamma and increased levels of receptor proteins and excitation. This study brings together human fMRI and animal research and enables improved therapeutic strategies for ameliorating pain and pain-related affective processing.

## Introduction

Pain, which is a negative experience involving sensory, emotional, cognitive and social dimensions, is classified into physical and social pain, which is defined as the painful feelings following social rejection, exclusion, or loss (Eisenberger, 2015; Williams and Craig, 2016). Growing evidence suggests that the experience of social pain relies on some of the same neurobiological processes underlying experiences of physical pain (Eisenberger, 2012). Pelvic pain, such as primary dysmenorrhea (PDM) which is menstrual pain without organic causes affecting approximately half of menstruating females (Iacovides et al., 2015), contains both acute and chronic components of pain (Wei et al., 2016) associated with impaired sensory and affective processes (Rhudy and Bartley, 2010), and structural and functional brain alterations (Low et al., 2018). It is becoming increasingly clear that PDM results in aberrant processing of physical and social pain (Pitangui et al., 2013; Yu et al., 2018). Interactional processing of physical and social pain (Borsook and MacDonald, 2010) involves similar brain regions, including the anterior cingulate cortex (ACC) and the adjacent medial prefrontal cortex (Bliss et al., 2016). Chronic pain causes increased neuronal activity in the ACC in humans (Hutchison et al., 1999), non-human primates (Iwata et al., 2005), and rodent pain models (Zhang et al., 2017), and is accompanied by neurophysiological and psychological changes (Bushnell et al., 2013), including depression and anxiety (Zhuo, 2016). Negative emotional stimuli, such as social exclusion (social pain), activate a range of brain areas involving the ACC (Eisenberger et al., 2003). Together, the above studies linked increased ACC neuronal activity to negative experience, highlighting the role of the ACC in processing and even potentially integrating physical and social pain. However, how pelvic pain, such as PDM, alters ACC-related neuronal pathways involved in the processing of neurophysiological and psychological stressors has not been tested in detail.

A key feature of chronic pain is the amplified affective response to nociceptive inputs (Zhou et al., 2018). The ACC processes and regulates both the sensory and affective component of pain (Eisenberger, 2012; Wager et al., 2016) and the hippocampus (HIPP) has been shown to participate in the integrative processing of pain (Bushnell et al., 2013). In particular, neuropathic pain alters HIPP-mediated behavior, synaptic plasticity and neurogenesis in rodents (Mutso et al., 2012). Moreover, the HIPP is involved when pain moves from an acute toward a chronic state, indicating a shift in the representation of pain in the brain from nociceptive to emotional circuits (Hashmi et al., 2013). Furthermore, the ACC interacts with the HIPP to mediate both cognitive and affective components of pain (Bliss et al., 2016). Increased activation of the ACC and HIPP is observed in post-traumatic stress disorder during the encoding of negative words (Thomaes et al., 2013). These findings implicate a critical role for both ACC and HIPP in the processing and integration of physical and social pain (Bliss et al., 2016; Jiang et al., 2018).

Functional connectivity (FC) between distant brain areas reflects neuronal and synaptic communications for the entrainment of various cognitive, emotional and sensory processing (Friston, 1994; Arieli et al., 1996; Tobia et al., 2017). Oscillatory activities, and their interplay, such as theta and gamma rhythms, render neuronal communication effective, precise, and selective, of which oscillatory coherence functions as a general indicator of communication between brain areas (Fries, 2015). Low frequency neural oscillations reflect large-scale network-level coordination across different neural circuits (Buzsaki and Draguhn, 2004). A recent human study highlighted the possible role of a disturbed dynamic coordination of the brain network in the pathophysiology of PDM and revealed abnormal low frequency theta oscillations in physical and social pain processing areas of the brain, such as the insula, parahippocampal gyrus, and cingulate cortex of PDM females (Lee et al., 2017). On the other hand, gamma oscillations represent the neuronal coordination of different brain regions (Gregoriou et al., 2009). Recent animal and human studies provide converging evidence that gamma oscillations are closely related to pain perception (Hu and Iannetti, 2019; Tan et al., 2019). In addition, cross-frequency phase-amplitude coupling (PAC), in which the amplitude of higher (e.g., gamma) rhythms is particularly modulated by the phase of lower (e.g., theta) rhythms, indicates a more complex regulatory feature through interactions between different frequency bands, such as long-range theta and local gamma communication (Wirt and Hyman, 2019; Chen et al., 2021). However, the possible abnormalities induced by PDM in ACC-HIPP connectivity and the underlying mechanisms of the condition remain unclear.

To address these issues, we employed integrative methods, including resting-state and task state (social exclusion task) functional magnetic resonance imaging (fMRI) in PDM humans, in combination with pharmacological, *in vivo* and *in vitro* electrophysiological, biochemical, and behavioral techniques in a pelvic pain rat model. We hypothesize that pelvic pain, such as PDM, may cause abnormal neuronal activity in the ACC and HIPP, and subsequent disruption in the connectivity between the two structures. The results obtained improve our understanding of how pelvic pain, including PDM, causes neural circuit changes and reveal that brain networks known to modulate both physical and social pain might display markers of central nervous system (CNS) abnormality in pelvic pain. The study provides some new preliminary support for the use of cross-species experiments to investigate pelvic pain, which may facilitate the search for relevant treatments.

## Materials and Methods

### Participants

This study was approved by the South China Normal University and Guangzhou University Institutional Ethics Review Board (2017–139). Human participants provided written informed consent prior to participation. A total of eighty right-handed (Li et al., 2017; Wasylyshyn et al., 2018) university female students (ages 18–25) who came to the recruitment were enrolled in this study which included 38 PDM and 42 non-PDM controls. PDM and control subjects were matched according to gynecological age. Demographic and clinical information are shown in Table 1. College students with dysmenorrhea (DM) were selected and PDM was further diagnosed in Sun Yat-sen Memorial Hospital, where magnetic resonance imaging (MRI) was performed to ensure there was no macroscopic structural abnormality inside or outside the uterus. Briefly, the diagnostic criteria for PDM were similar to those defined by the American College of Obstetricians & Gynecologists (American College of Obstetricians and Gynecologists, 2006). The following inclusion criteria were used for PDM participants: (1) a menstrual cycle of average 30 days; (2) a history of menstrual pain over more than 12 months; (3) a self-assessed severity of the average menstrual pain of 5 and above over the previous 6 months based on the visual analog scale (VAS, 0 = not at all, 10 = the worst imaginable pain); and (4) a pelvis MRI scan did not show any anatomical pelvic disease. The inclusion criteria for the controls were similar to those for the PDM subjects except that the controls had a self-assessed VAS of 0. Exclusion criteria included pregnancy, organic pelvic disease, alcohol or drug abuse, failure of MRI scans due to metal or pacemaker implants, and formal diagnosis of psychiatric conditions. Urinary luteinizing hormone tests were performed to verify experimentally whether the participants were in their periovulatory phase (i.e., days 12–16 of the menstrual cycle), which is the phase when influence of chronic PDM was usually evaluated (Wei et al., 2016; Liu J. et al., 2017; Liu et al., 2018).

**TABLE 1:** Demographic and clinical information of the PDM subjects and controls used in human MRI.

	Resting-state fMRI (M ± SD)			Task fMRI (M ± SD)			Structural MRI (M ± SD)		
			
	PDM (n = 35)	Ctrl (*n* = 38)	*p* value	PDM (n = 30)	Ctrl (*n* = 31)	*p* value	PDM (n = 38)	Ctrl (*n* = 42)	*p* value
Age, years^*a*^	20.49 ± 1.20	20.58 ± 1.52	0.694	20.70 ± 1.12	20.45 ± 1.06	0.326	20.55 ± 1.22	20.60 ± 1.47	0.633
Age of onset of menstruation, years^*a*^	12.60 ± 1.31	13.05 ± 1.51	0.196	12.53 ± 1.31	12.32 ± 2.75	0.952	12.63 ± 1.28	12.95 ± 1.50	0.364
Menstrual duration, years^*a*^	7.89 ± 1.75	7.53 ± 1.70	0.417	8.17 ± 1.76	8.13 ± 2.90	0.479	7.92 ± 1.71	7.64 ± 1.66	0.551
Menstrual cycle, days^*a*^	30.63 ± 2.77	29.13 ± 2.53	0.019	29.67 ± 3.16	28.74 ± 2.62	0.120	30.50 ± 2.82	29.17 ± 2.51	0.023
Pain begin age, years	15.14 ± 1.99	N/A	N/A	14.93 ± 2.12	N/A	N/A	15.03 ± 1.98	N/A	N/A
Pain duration year, years	5.14 ± 2.13	N/A	N/A	5.53 ± 2.22	N/A	N/A	5.29 ± 2.12	N/A	N/A
Pain degree	6.54 ± 1.09	N/A	N/A	6.77 ± 1.03	N/A	N/A	6.61 ± 1.10	N/A	N/A
Positive emotion									
Pre-test^*a*^	N/A	N/A	N/A	15.03 ± 5.93	16.45 ± 5.54	0.188	N/A	N/A	N/A
Post-test^*a*^	N/A	N/A	N/A	13.13 ± 4.96	14.71 ± 5.69	0.304	N/A	N/A	N/A
*p*-value within group^*b*^	N/A	N/A	N/A	0.088	0.059	N/A	N/A	N/A	N/A
Negative emotion									
Pre-test^*a*^	N/A	N/A	N/A	12.23 ± 2.54	11.39 ± 2.35	0.151	N/A	N/A	N/A
Post-test^*a*^	N/A	N/A	N/A	12.67 ± 3.17	12 ± 3.54	0.155	N/A	N/A	N/A
*p*-value within group^*b*^	N/A	N/A	N/A	0.771	0.297	N/A	N/A	N/A	N/A
BNQ									
Pre-test^*c*^	N/A	N/A	N/A	2.33 ± 0.42	2.42 ± 0.40	0.407	N/A	N/A	N/A
Post-test^*c*^	N/A	N/A	N/A	3.11 ± 0.42	3.10 ± 0.46	0.988	N/A	N/A	N/A
*p*-value within group^*d*^	N/A	N/A	N/A	<0.001	<0.001	N/A	N/A	N/A	N/A

*PDM, primary dysmenorrhea; Ctrl, controls; BNQ, basic needs questionnaire. ^a^Mann–Whitney test; ^b^Wilcoxon test; ^c^Two-sample t-tests; ^d^Paired-sample t-tests; M, mean value; SD, standard deviation; N/A, non-applicable.*

### Human Experimental Procedure

The scheme of the experiment is presented in Figure 2A. After arriving at the lab, participants were asked to complete a detailed consent form. Psychological questionnaires (including the Positive and Negative Affect Scale, PANAS (Watson et al., 1988), and the Basic Needs Questionnaire, BNQ) (Bernstein and Claypool, 2012) were processed to obtain the baseline emotional state. Next, participants with PDM underwent abdominal and pelvic cavity, uterus and accessory MRI scans, and clinicians made the final diagnosis on whether the subjects were PDM patients. Participants who met the inclusion criteria then read the instructions outside the MRI room, and the researcher orally interpreted the instructions to the participants, indicating the duration of the experiment, the requirement to keep the head fixed and to warn about the noise of the machine and related equipment. Then, groups of three participants (including one real participant in the study and two fixed female ‘actors’) engaged in a 10-min group interaction session, after which participants were told to begin a Cyberball game, a paradigm based on a virtual ball-tossing game, where participants believe they are playing with other real participants, although in fact these are computer-generated (Eisenberger et al., 2003). Blood oxygenation level-dependent (BOLD) signal changes were recorded during the Cyberball task.

In the scanner, participants saw an animated ball-tossing game, with an icon representing their own hand at the bottom and the two other players depicted as animated icons in the upper corners. The names of the group members were shown next to each icon and participants could throw the ball to whoever they liked. Pressing “1” delivers the ball to the member in the upper left corner, while pressing “2” throws the ball to the member in the upper right corner. Participants were instructed to throw the ball within 2 s of receiving it. If the time exceeds 2 s, the system will temporarily transfer the ball randomly. The computer players waited 0.5–2.0 s before making a throw to heighten the sense that the participant was actually playing with other individuals. Each participant participated in three rounds of the Cyberball game during three fMRI scans. Each round of the Cyberball program consisted of 60 throws (including participant and computer players’ throws) and lasted 3 min. The three rounds of Cyberball (Figure 2B) were as following, (1) Cyberball observation, implicit social exclusion (ISE), where participants were told that the intranet connection was not effective yet because of technical problems, but that they could watch the other participants playing; (2) Cyberball inclusion (INCL), where participants were told they were connected and played with the other players, participant and other players were equally likely to throw the ball; and (3) Cyberball exclusion, explicit social exclusion (ESE), where participants received three throws and were then excluded from the game (i.e., the other players started playing exclusively together, and the real participant never received the ball again) (Eisenberger et al., 2003).

Participants and experimenters were in a double-blind state, namely, participants were told that the purpose of the study was to examine the effect of imagination on mission performance; the experimenter did not know who among the participants was a PDM subject. Immediately following the scanning session, participants completed the PANAS and BNQ questionnaires again. At the end of the experiment, participants were asked whether they believed they were playing the Cyberball game with the other two participants (i.e., the ‘actors’ they met initially). Subjects who failed to initiate social exclusion were excluded according to their answers (for example, if they did not believe that they were playing a real game of pitching, but thought it was a pre-set experimental procedure) and the PANAS and BNQ scales scores before and after the Cyberball game were compared (Eisenberger et al., 2006). In this study, both PDM subjects and controls showed a significantly higher post-BNQ score than pre-BNQ score, indicating that the participants noticed the exclusion and felt excluded (Table 1). Finally, each participant received 70 yuan (RMB) as compensation and was thoroughly debriefed about the purpose of the study.

### MR Data Acquisition and Preprocessing

Magnetic resonance (MR) data were acquired on a 3.0 Tesla clinical scanner (Achieva TX; Philips Healthcare, Best, Netherlands) with an 8-channel head coil in Sun Yat-sen Memorial Hospital, Sun Yat-sen University. We performed resting-state fMRI (rs-fMRI) before task fMRI (T-fMRI) using a T2^∗^-weighted fast-field echo-planar imaging (FFE-EPI) sequence (rs-fMRI/T-fMRI TR = 2000 ms/3000 ms, TE = 30 ms, FA = 90°, FOV = 240 mm × 240 mm, acquisition matrix = 64 × 64, thickness = 4.0 mm, 33 transverse slices covering the whole brain; 240/60 volumes were obtained for rs-fMRI/each round of T-fMRI). High-resolution structural images were collected using a T1-weighted 3D FFE sequence (TR = 8.2 ms, TE = 3.7 ms, FA = 8°, FOV = 256 × 256, acquisition matrix = 256 × 256, thickness = 1 mm; 168 sagittal slices covered the whole brain).

Rs-fMRI data were preprocessed using SPM 12^1^ and the DPABI v3.1 toolbox^2^ in MATLAB. For each subject, the first 10 functional images were discarded to reach magnetization equilibrium and to allow adaptation to the MR environment. Then, a slice-timing correction was conducted by setting the middle slice (17th) as the reference. Realignment was performed to estimate head motion, and two PDM subjects were excluded due to excessive head motion (translation more than 2 mm, or rotation more than 2°). We also calculated the mean frame-wise displacement based on Jenkinson’s model (FD-Jenkinson), and ensured there was no significant group effect on the FD-Jenkinson (Jenkinson et al., 2002). High-resolution structural images were co-registered into functional images and segmented into white matter, gray matter and cerebrospinal fluid. Then, we spatially normalized the functional images to the individual structural image in standard MNI-152 standard space with a resampled voxel size of 3 mm × 3 mm × 3 mm; one PDM and four healthy subjects were excluded due to bad normalization (such as, no alignment between functional image and MNI-152 template; functional signal loss of normalization map). Furthermore, nuisance covariates (Friston 24 head motion parameters, mean white matter and mean cerebrospinal fluid) were regressed out to reduce the effect of complex noise. Finally, we further conducted spatial smoothing with a Gaussian kernel of 4 mm full-width at half maximum (FWHM) and performed band-pass filtering (0.01–0.1 Hz) to reduce high-frequency physiological noise. Finally, 35 PDM subjects and 38 controls were included in the further rs-fMRI analysis.

In the T-fMRI, 34 PDM subjects and 36 controls completed the three rounds of Cyberball. For each round of this task, the first five volumes were removed to control for interference between rounds. Other T-fMRI preprocessing steps, including slice-timing, realignment, co-registration, normalization and spatial smoothing, were conducted as for rs-fMRI preprocessing. A high-pass filter (cutoff 128 s) was used to remove low-frequency noise. After quality control, nine subjects with excessive head motion or bad normalization were excluded, leaving 30 PDM subjects and 31 healthy controls for further analysis.

### Static FC Between ACC and HIPP in the rs-fMRI

**FIGURE 1:**
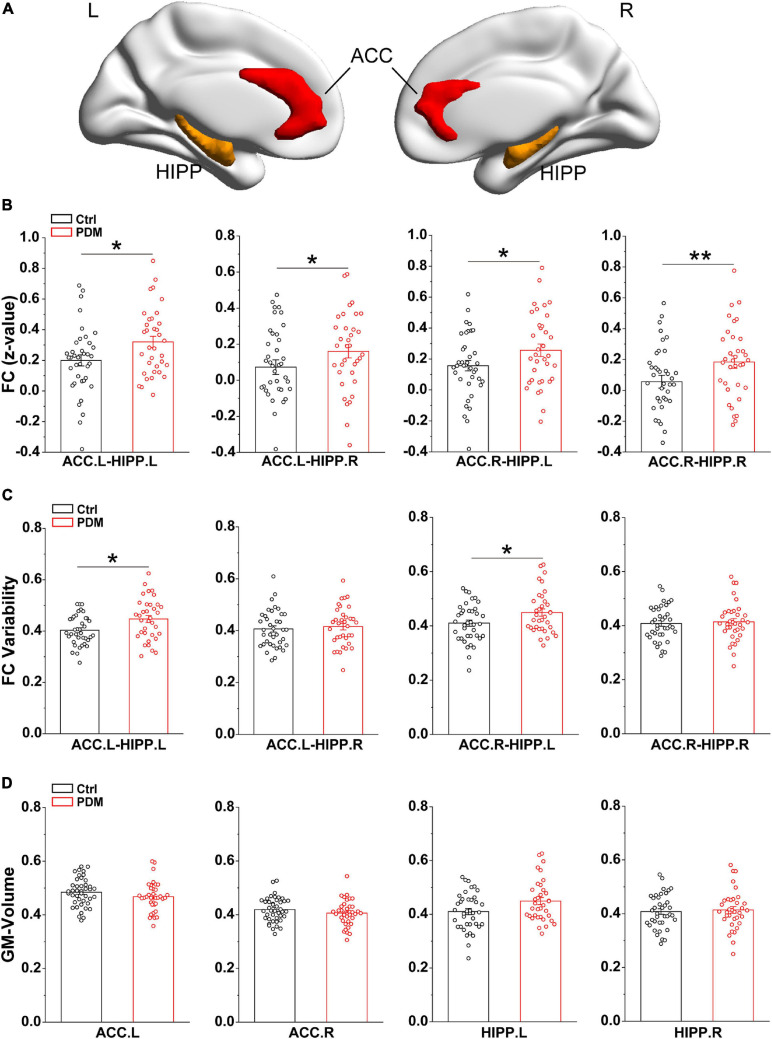
Greater static FC and FC variability between ACC and HIPP, with unchanged GM volumes, in PDM females. **(A)** Three-dimensional brain view of the ICBM152 MNI depicting the location of the bilateral ACC (red) and HIPP (yellow) based on the Anatomical Automatic Labeling (AAL) atlas. **(B)** In the static FC, PDM subjects exhibited increased FC between ACC.L-HIPP.L, ACC.L-HIPP.R, ACC.R-HIPP.L, and ACC.R-HIPP.R. **(C)** The PDM group exhibited significantly greater FC variability between ACC.L-HIPP.L and ACC.R-HIPP.L than controls. **(D)** The GM volume of both ACC and HIPP did not change significantly in PDM females compared to controls (*p* > 0.05). L, left hemisphere; R, right hemisphere. Error bars = ± 1 SE. *n* = 35 (38) and 38 (42) for PDM and control females in the static FC, FC variability (GM volume) analysis, respectively. Non-parametric permutation test with days of menstrual cycle as regressor for static FC, FC variability and GM volume. ^∗^*p* < 0.05; **p < 0.01.

Since static FC assumes that brain connectivity is temporally stationary (Fox and Raichle, 2007), we calculated the correlation coefficient between ACC and HIPP over the whole scan time to reflect the overall connections. The bilateral ACC and HIPP were selected as regions of interest (ROIs) for ROI-based FC based on the Automated Anatomical Labeling atlas (AAL) (Rolls et al., 2015), which is widely used for human brain imaging analysis. The specific MNI locations of the ROIs (ACC and HIPP) are viewed on the ICBM152 human brain surface (Mazziotta et al., 2001; Figure 1A). For each participant, we extracted the time courses of each ROI from preprocessed images. Then, the Pearson’s correlation coefficients (*r*) between ACC and HIPP were computed. To improve the normality of group analysis, Fisher’s *z*-transformation was performed to convert the *r*-value into a *z*-value.

### Dynamic FC Between ACC and HIPP in the rs-fMRI

Dynamic FC between ACC and HIPP was calculated using a sliding time-window approach. We fixed the length of the time-window at 20 TRs (40 s) and the sliding step to 1 TR: 211 FC matrices between ACC and HIPP were obtained for each subject [time points: TP = 230 TRs; length of time-window (L) = 20 TRs; sliding step = 1 TR; dynamic FC matrices: *T* = *TP* – *L* + 1 = 211] (Li et al., 2014). The standard deviation of FC across all 211 slide-window FC matrices was calculated as FC variability, which reflects the discreteness of FC.

### GLM and PPI Analysis in the T-fMRI

Whole-brain general linear model (GLM) analysis was performed using standard hemodynamic response function to identify brain activation during the Cyberball task. Each round of Cyberball (ISE, INCL, ESE) was modeled using a block design. The contrasts of interest – implicit exclusion compared with inclusion (ISE-INCL), explicit exclusion compared with implicit exclusion (ESE-ISE), and explicit exclusion compared with inclusion (ESE-INCL) – were then computed to depict social exclusion resulting from peer rejection. The GLM parameter maps of the contrasts were generated for each participant. Significant differences in brain activation were evaluated by performing a group comparison of the GLM-parameter maps.

Psychophysiological interaction (PPI) analysis (Friston et al., 1997) was conducted to determine which cerebral regions were functionally connected with the ROI for each of the ISE-INCL, ESE-ISE, ESE-INCL contrasts separately. Prior studies suggested that the ACC is activated by social exclusion (Bolling et al., 2011; Masten et al., 2011) and therefore the ACC (ACC.L/ACC.R/combined bilateral ACC) was chosen to initiate the PPI analysis. The deconvolved activity time-series of the left ACC, right ACC, and combined bilateral ACC were extracted and adjusted for effects of interest (ISE-INCL, ESE-ISE, ESE-INCL), and the PPI term was created using the ROI eigenvariate and the specific task contrasts (ISE-INCL, ESE-ISE, ESE-INCL). Finally, a second GLM was performed with a condition-specific regressor probing each contrast (ISE-INCL, ESE-ISE, ESE-INCL) to obtain the PPI parameter map, allowing the identification of ACC-connectivity changes for social exclusion during Cyberball. To further clarify the connectivity between ACC and HIPP under social exclusion conditions, the mean PPI parameter of HIPP was extracted for the group comparison.

### Voxel-Based Morphology Analysis of the Structural MRI

The T1-weighted structural images were preprocessed with the VBM toolbox in the SPM12. The GM volume of each subject was evaluated using voxel-based morphology (VBM) analysis. First, the structural image was manually reoriented to MNI space and centered on the anterior commissure to facilitate the following segmentation step. The reoriented image was then segmented into GM and white matter using the unified segmentation approach. Spatial smoothing with 4 mm FWHM was performed on the warped and modulated GM map to improve the spatial resolution. Finally, we restricted our search regions to the ACC and HIPP, rather than the whole brain. To achieve this, we extracted the GM signal of the ACC and HIPP for each subject.

### Experimental Animals

Female Sprague-Dawley (SD) rats were purchased from the Laboratory Animal Center of Southern Medical University (Guangzhou, China) and kept at the School of Life Sciences, South China Normal University, with controlled humidity and temperature, and a 12 h (6:30 AM to 6:30 PM) light–dark cycle. Rats involved in experiments were used according to international and university ethical standards. Food and water were available *ad libitum*. Animals weighing 250–300 g (120–150 days old) were given on average 7 days to adjust to the new environment prior to the experiments. Pelvic pain model was generated by intraperitoneal (IP) injection of estradiol benzoate and oxytocin (both Ningbo Hormone Inc., China).

### Rat PDM Model

The experimental procedures of this study were approved by the Animal Protection and Use Committee of Guangzhou University and South China Normal University. The chronic PDM rat model was generated by modification of an acute PDM mouse model (Chen et al., 2013; Jesuino et al., 2019). Briefly, estradiol benzoate was injected (IP, two times/week, 4 mg/Kg) for eight consecutive weeks (wks). From the fourth wk through the eighth wk, PDM rat model were injected with oxytocin (IP, one injection/week, 20 IU/Kg/per injection) 24 h after injection of estradiol benzoate. Control rats received injections of an equal volume of estradiol benzoate and saline. Paw withdrawal mechanical threshold (PWMT) (Chaplan et al., 1994; Liu Y. et al., 2017), electrophysiological and biochemical evaluations were conducted in control and PDM rats between 24 and 72 h following the last oxytocin injections.

### *In vivo* Surgery and Extracellular Recording

*In vivo* dual-site extracellular recordings were conducted as described with a few modifications (Noguchi et al., 2017; Chen et al., 2019). Rats were anesthetized with pentobarbital sodium (IP 80 mg/kg, Sigma, United States) then head-fixed in a stereotaxic apparatus (RWD Life Science, China) with body temperature maintained between 36 and 37°C. When necessary, a supplemental dose of anesthesia was given based on tail reflex. After a midline skin incision was made, two skull holes were drilled above the ACC (2.5 mm anterior to the bregma, 0.4 mm lateral to the midline, 1.7-2.0 mm depth) and the dorsal CA1 subregion of the HIPP (3.6 mm posterior to the bregma, 2.0 mm lateral to the midline, 2.2–2.5 mm depth, 10°) under a stereomicroscope (Sunny Optical Technology, China). Two glass microelectrodes for recording (filled with 0.5 M NaCl, resistance 4–6 MΩ) were slowly inserted until the tips of the electrodes reached the ACC and hippocampal CA1. Each recorded signal was amplified (1,000x) by an electrometer amplifier (Model 3000; A-M Systems, United States) and digitized via a D/A converter (Micro 1401; Cambridge Electronic Design, Ltd., United Kingdom), then sent to data acquisition software (Spike2; Cambridge Electronic Design).

### LFP Analysis

Extracellular recording data were analyzed offline in MATLAB. For processing the local field potential (LFP), a Butterworth low pass filter (300 Hz) was applied to the raw recorded data. Power spectral density was computed using Thomson’s multitaper method for a fast Fourier transformation (FFT) to determine the power for specific frequency bands. Frequency ranges were defined as follows: delta: 1–4 Hz; theta: 4–12 Hz; gamma: 30–100 Hz, of which theta frequency covers a wider range in the rodent (Buzsaki and Draguhn, 2004; Tamura et al., 2017).

We performed synchronization analysis in line with our established methodological protocol (Chen et al., 2019). Simultaneous signals were subjected to cross-correlation estimation to quantitatively evaluate the similarity. The maximal offset was set to ±1 s. After the calculation, spectral coherence between the two LFPs from the ACC and HIPP was analyzed using a FFT number of 2^12^, and the values ranged from 0 to 1, meaning non-correlated or completely correlated in the frequency domain.

To further evaluate the synchrony between the oscillations of ACC and HIPP, weighted phase lag index (WPLI) analysis, which is based on the complex conjugate of spectral coherence (Vinck et al., 2011), was conducted to the same dataset. The indices were shown from 0 to 1 as mentioned above.

To access the modulation strength of cross-frequency oscillations, we first derived the instantaneous phase and amplitude from the targeted signals of both areas, then analytically clustered the theta phases binned into 20° intervals with the corresponding gamma amplitude at the same time in the other region. Both directions were analyzed to compare the influence of the theta band on the interregional gamma band.

### Whole-Cell Patch-Clamp Recording

Acute brain slices containing ACC and HIPP (350 μm) were prepared according to routine procedures (Chen et al., 2017; Luo et al., 2019), from control and PDM rats using a vibratome (VT 1000S, Leica, Germany) in oxygenated ice-cold cutting solution containing (in mM), 119 NaCl, 2.5 KCl, 2.5 CaCl_2_,1.3 MgSO_4_, 1 NaH_2_PO_4_, 11 D-glucose, 26.2 NaHCO_3_ (pH 7.2-7.4), saturated with 95% O_2_/5% CO_2_. Slices were kept in artificial cerebrospinal fluid (aCSF) containing (in mM) 140 NaCl, 4.7 KCl, 2.5 CaCl_2_, 1.2 MgCl_2_, 11 D-glucose, 10 HEPES (pH 7.2-7.4), and gassed with 95% O_2_/5% CO_2_. Slices were incubated for 1 h at 30–32°C before recording and then transferred to a submerged recording chamber where temperature was held at 32 ± 0.5°C with an automatic temperature controller (TC-324B, Warner Instrument Corporation) with aCSF flow set at 2–3 ml/min.

Pyramidal neurons were identified by their morphology, typically characterized by a triangular-shaped soma, in brain slices (Ramaswamy and Markram, 2015). To record miniature excitatory and inhibitory postsynaptic currents (mEPSCs and mIPSCs) from pyramidal neurons of the ACC and HIPP, voltage was held at –60 and 0 mV, respectively. To block fast sodium channel activity and thus action potential, 1 μM TTX was added to the aCSF (Chen et al., 2017). The pipette was filled with the following internal solution (mM): 100 mM Cs-gluconate, 5 mM CsCl, 10 mM HEPES, 2 mM MgCl_2_, 1 mM CaCl_2_, 11 mM BAPTA, 4 mM ATP, and 0.4 mM GTP (pH 7.3, adjusted with KOH) at an osmolality of 280–290 mOsm. Data were collected with a MultiClamp 700 B amplifier (Axon Instruments) and filtered during acquisition with a low pass filter set at 2 kHz using pCLAMP10 software (Molecular Devices, United States). The data were analyzed offline using Mini Analysis Program (Synaptosoft Inc., United States).

### Western Blotting Analysis

Rat brains were dissected and ACC and HIPP tissues were removed on ice as previously described (Chen et al., 2017). Tissues were then homogenized in SDS buffer (50 mM Tris pH 7.5, 150 mM NaCl, 5 mM EDTA pH 8.0, 1% SDS). Cellular debris was removed by centrifugation at 4°C (14,000 rpm for 10 min) and the supernatant was collected for analysis. Tissue lysates were subjected to SDS-PAGE, and transferred to nitrocellulose membranes. The membranes were blocked with 5% non-fat dry milk and incubated with specific primary antibodies GluR1 (Abcam, ab31232; dilution 1:1000), GluR2 (Abcam, ab206293; dilution 1:2000), GluR4 (Abcam, ab119995; dilution 1:4000), NMDAR1 (Abcam, ab109182; dilution 1:4000) or NMDAR2B (Abcam, ab65783; dilution 1:4000) overnight at 4°C. GAPDH (Beyotime, AF0006; dilution 1:5000) or anti-β-actin antibody (Sigma, United States; dilution 1:5000) was used as a loading control. After three washes with TBST, HRP-labeled secondary antibody (CWS, China) was added at room temperature for 1 h using 5% milk in TBST followed by three additional washes with TBST. The Immobilon ECL western system (Millipore, United States) was then used to visualize the bands, which were quantified and analyzed with Gel-Pro Analysis software (Media Cybernetics, United States).

### Statistical Analysis

Normal distribution was tested in demographics and psychological data. Except for the BNQ score, all data were not distributed normally. Thus, Mann–Whitney tests were used to detect differences in age, age of onset of menstruation, menstrual duration, menstrual cycle, and PANAS between the PDM subjects and healthy controls. Wilcoxon test was used to detect difference in PANAS between pre- and post-tests. Parametric tests (including two-sample *t*-tests and paired-sample *t*-tests) were performed to determine differences in BNQ between and within group, respectively. Two-sample *t*-tests were also performed to detect group differences in the GLM-parameter map and PPI-parameter map, to identify abnormal regional brain activity and abnormal regions functionally connected with the ACC. Multiple comparison corrections were conducted using an AlphaSim correction (both voxel-wise threshold and cluster threshold were set as *p* < 0.05) and the Gaussian Random Field correction (voxel-wise threshold: *p* < 0.01; cluster threshold: *p* < 0.05) separately. A non-parametric permutation test was conducted to identify between-group differences in the static FC, FC variability, GM volume and ACC-HIPP PPI parameters. In the calculation, we took the menstrual cycle as covariate and regressed it out if there was a significant difference in the menstrual cycle between PDM subjects and controls. For LFP analysis, the data were first tested for normal distribution. None of the datasets were distributed normally, therefore a non-parametric test was used for two-group comparisons. Student’s *t*-test was used in two-group comparisons of western blotting results. For comparisons of multiple groups, one-way ANOVA or two-way ANOVA with *post hoc* tests were used. Data are shown as mean ± SEM unless otherwise stated. Statistical significance threshold was set at *p* < 0.05.

## Results

### Demographic and Clinical Information of PDM Subjects and Controls Used in MRI

For the rs-fMRI and structural MRI, no significant differences were found for the age, age of onset of menstruation or menstrual duration between the PDM subjects and controls, while a longer menstrual cycle was found in the PDM subjects. For the T-fMRI, there were no significant between-group differences for age, age of onset of menstruation, menstrual duration, menstrual cycle, PANAS, and BNQ. Both groups showed no significant difference in PANAS between pre- and post-test values, and a significantly higher post-BNQ score than pre-BNQ score. The specific values are presented in Table 1.

### Significantly Increased FC in rs-fMRI Between ACC and HIPP in PDM Women

To examine the synchronization of blood oxygenation level dependent (BOLD) signals between the ACC and HIPP, we first evaluated FC, utilizing rs-fMRI, for both static and dynamic FC in 35 PDM women with 5.14 ± 2.13 years dysmenorrhea and 38 age-matched controls (Table 1). Static FC increased significantly in PDM subjects between left/right ACC and left/right HIPP (ACC.L-HIPP.L, ACC.L-HIPP.R, ACC.R-HIPP.L, ACC.R-HIPP.R) compared to controls (*p* < 0.05; Figure 1B), indicating that experiencing 5 years of PDM changes brain FC between the ACC and HIPP. Given that the human brain is a complex and interactive system that dynamically processes information flow and that changes in FC over time are not revealed by static FC evaluations (Hutchison et al., 2013), we next examined the variability in FC, i.e., changes in the connections between the ACC and HIPP, using a sliding time window (see section “Materials and Methods” for details). The dynamic FC calculations revealed significantly increased FC variability between the left ACC and left HIPP (ACC.L-HIPP.L) as well as between the right ACC and left HIPP (ACC.R-HIPP.L) in the PDM cohort (*p* < 0.05; Figure 1C). Thus, both static and dynamic FC suggest an overall increase in communication between the ACC and HIPP in PDM females.

### Alterations in FC Revealed by T-fMRI During Social Exclusion Induced by Cyberball

Given that being socially integrated is a primary human need (Adolphs, 2010), and that emotional/mental stimuli can lead to altered activation of brain areas, such as the ACC (Singer et al., 2004), we next asked whether the experience of PDM, a type of physical pain, would change the brain activation and FC when dealing with negative and positive emotional stimulations, i.e., social exclusion and inclusion conditions. To do so, we used the Cyberball task, a paradigm based on a virtual ball-tossing game, including three scenarios of implicit social exclusion (ISE), inclusion (INCL), and explicit social exclusion (ESE) (Williams et al., 2000; Eisenberger et al., 2003).

**FIGURE 2:**
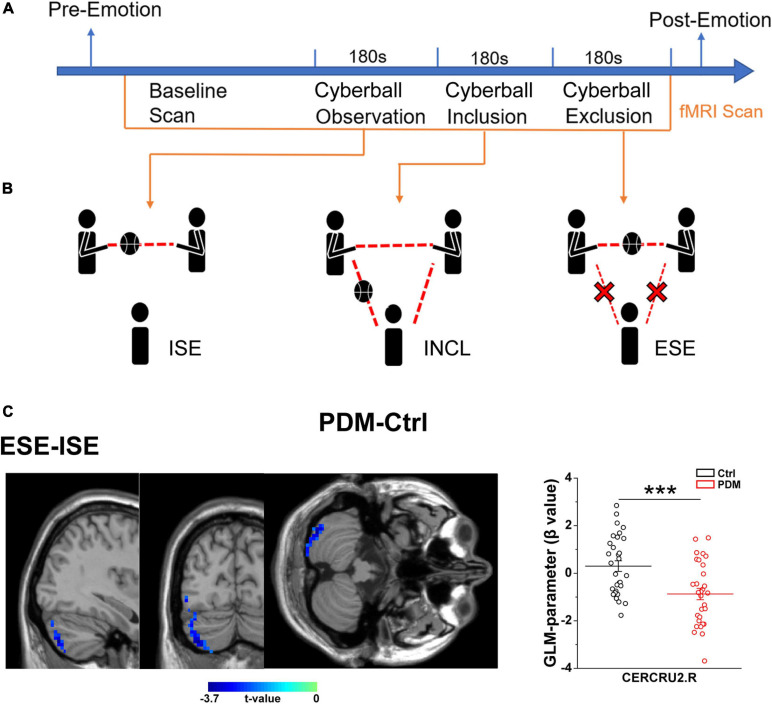
Abnormal brain activation in the right CERECRU2 for the ESE-ISE contrasts in PDM females. **(A)** Scheme of the experiment: baseline emotional state was collected using psychological questionnaires including Positive and Negative Affect Scales (PANAS) and Basic Needs Questionnaire (BNQ) for each subject. Then, MRI scans were performed in the resting-state and during three Cyberball scenarios. Finally, the PANAS and BNQ were conducted again to record the post-emotional status of the subjects. **(B)** Schematic representation of the three Cyberball scenarios: Cyberball observation represents ISE, where participants were told that the intranet connection was not yet effective due to technical issues, but that they could watch other participants play; Cyberball inclusion, INCL, where subjects participated in the social activity of passing the ball; Cyberball exclusion, ESE, individuals were prevented from participating in the social activity of passing the ball by other players playing among themselves only. **(C)** Significantly reduced activation in the right CERCRU2 for the ESE-ISE contrasts in the PDM group compared to Controls. The GLM-parameter (β value) for each region was extracted to reflect the brain activation in the PDM and Controls. *n* = 30 and 31 for PDM and control females, respectively. Error bars = ± 1 SEM. Two-sample *t*-test and AlphaSim correction with voxel-wise threshold of *p* < 0.05 and a cluster threshold of *p* < 0.05 were used to determine the brain regions with significantly abnormal activity. The color bar represents the *t*-value. CERCRU2, Crus II of cerebellar hemisphere; R, right hemisphere. ***p < 0.001.

We first evaluated the condition-related differences in regional brain activity as measured by T-fMRI utilizing the general linear model (GLM), a method used to evaluate differences in activation under various conditions by subtracting one condition from another, i.e., in this case, ISE-INCL, ESE-ISE, and ESE-INCL (Friston et al., 1997). In the GLM analysis, PDM females showed decreased activation of the right Crus II of the cerebellar hemisphere (CERCRU2) in the ESE-ISE contrast compared to controls (Figure 2C and Table 2). No significant difference was found in the ISE-INCL and ESE-INCL contrast between PDM subjects and controls (Table 2).

**TABLE 2:** Specific brain regions show significant group differences in activation for ISE-INCL, ESE-ISE, ESE-INCL contrasts.

	Peak location (AAL-90)	No. of voxels	Peak *t*-value	Peak coordinate in MNI space			Included other regions	GLM-parameter (β value) (mean ± SEM)	
			
				X	Y	Z		PDM	Ctrl
ISE-INCL									
PDM-Ctrl	N/A	N/A	N/A	N/A	N/A	N/A	N/A	N/A	N/A
ESE-ISE									
PDM-Ctrl	CERCRU2.R	196	–3.655	36	–78	–48	CERCRU1.R, ITG.R	–0.873 ± 0.241	0.298 ± 0.224
ESE-INCL									
PDM-Ctrl	N/A	N/A	N/A	N/A	N/A	N/A	N/A	N/A	N/A

*ISE, implicit social exclusion; INCL, inclusion; ESE, explicit social exclusion; PDM, primary dysmenorrhea; Ctrl, controls; GLM, general linear model; MNI, Montreal Neurological Institute; AAL, automated anatomical labeling atlas; CERCRU2, Crus II of cerebellar hemisphere; CERCRU1, Crus I of cerebellar hemisphere; ITG, inferior temporal gyrus; SEM, standard error of mean; R, right hemisphere. N/A, non-applicable.*

**FIGURE 3:**
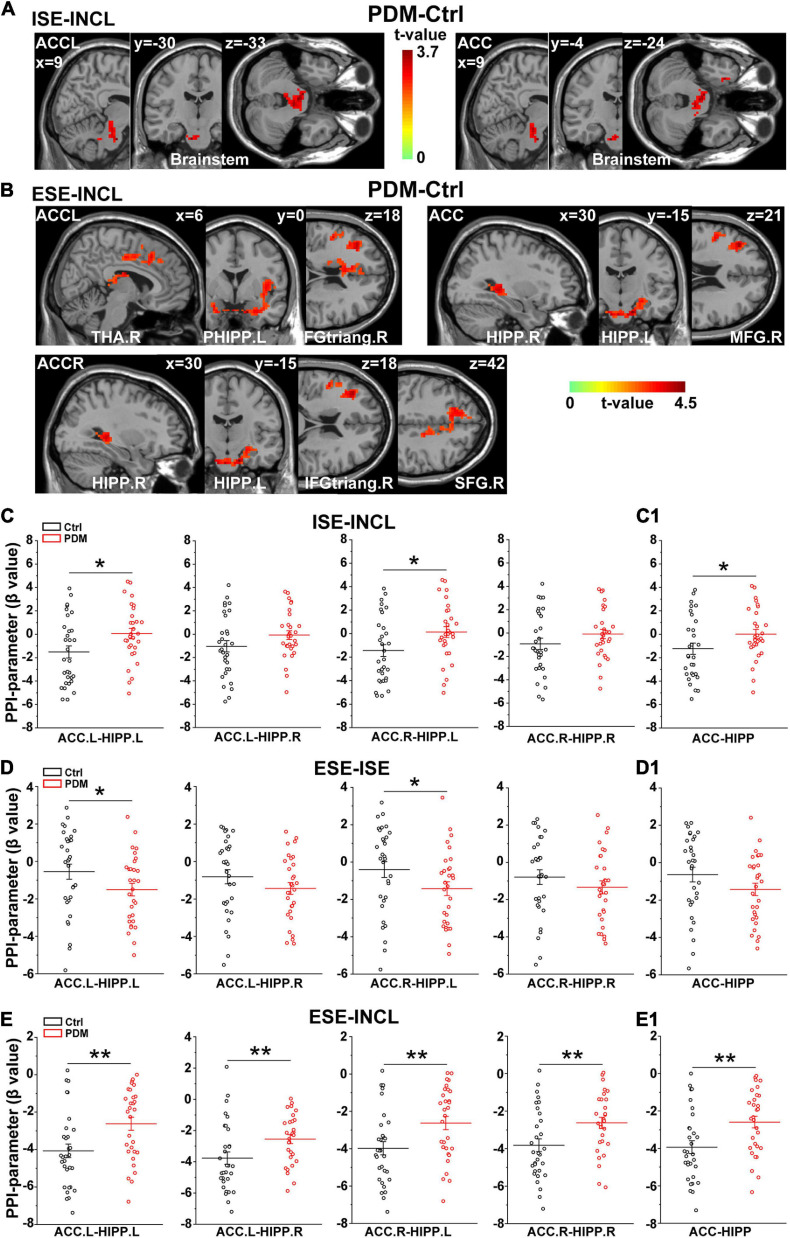
Abnormal connectivity between ACC and HIPP in PDM females revealed by PPI analysis. **(A,B)** The PDM cohort has increased ACC connectivity in the ISE-INCL and ESE-INCL contrasts. In the ISE-INCL contrast **(A)**, PDM females show increased ACC.L/ACC connectivity with brainstem. In the ESE-INCL contrast **(B)**, PDM women demonstrate increased connectivity between ACC.L/ACC.R/ACC and other brain regions, including frontal cortex, thalamus, and HIPP. **(C–E)** The PDM group exhibits a significant difference in ACC-HIPP connectivity compared to controls. For the ISE-INCL contrast **(C)**, PDM subjects show increased ACC.L-HIPP.L and ACC.R-HIPP.L connectivity. ACC.L-HIPP.R and ACC.R-HIPP.R connectivity is identical. In the ESE-ISE contrast **(D)**, the PDM group show decreased connectivity for the ACC.L-HIPP.L and ACC.R-HIPP.L circuits. ACC.L-HIPP.R and ACC.R-HIPP.R connectivity is also identical. In the ESE-INCL comparison **(E)**, PDM subjects demonstrate increased connectivity for all ACC-HIPP combinations. Combining the bilateral regions also shows that PDM women have an overall increased ACC-HIPP connectivity in the ISE-INCL contrast **(C1)** and the ESE-INCL contrast **(E1)**; and no significant difference in the ESE-ISE contrast **(D1)**. Given that the PPI value is negative, the increased ACC-HIPP connectivity in the PDM indicated less connectivity reduction in the ISE-INCL and ESE-INCL contrast. L/R, left/right hemisphere; *n* = 30 and 31 for PDM and control females, respectively. The color bar represents the *t*-value. Error bars = ± 1 SEM. Two-sample *t*-test and Gaussian Random Field (GRF) correction with voxel-wise threshold of *p* < 0.01 and cluster threshold of *p* < 0.05 were used to determine the significantly abnormal brain regions functionally connected with ACC.L/ACC.R/ACC. A non-parametric permutation test was used for the specific ACC-HIPP connectivity with *p* < 0.05. *p < 0.05; **p < 0.01.

We next conducted psychophysiological interaction (PPI) analysis (Friston et al., 1997) to determine which brain regions were functionally connected with the ACC. In the ISE-INCL contrast, the FC between ACC.L-brainstem and ACC-brainstem (including the right parahippocampal region, PHIPP.R) was higher in PDM females than in controls (Figure 3A and Table 3). In the ESE-INCL condition, PDM females demonstrated increased connectivity between ACC.L-right thalamus (THA.R)/PHIPP.L/right inferior frontal gyrus, triangular part (IFGtriang.R), ACC.R-HIPP.R/HIPP.L/IFGtriang.R/right superior frontal gyrus (SFG.R), and ACC-HIPP.R/HIPP.L/right middle frontal gyrus (MFG.R) (Figure 3B and Table 3). Identical FC between regions connected with ACC.L/ACC.R/ACC was observed in PDM subjects and controls in the ESE-ISE contrast. Together, the results indicate increased connections between the ACC and the above brain areas in response to Cyberball-induced social exclusion in PDM females.

**TABLE 3:** Specific brain regions show significant group differences in functional connectivity with ACC.L/ACC.R/ACC for ISE-INCL, ESE-ISE, ESE-INCL contrasts.

ROI	Peak location (AAL-90)	No. of voxels	Peak *t*-value	Peak coordinate in MNI space			Included other regions	PPI-parameter (β value) (mean ± SEM)	
			
				X	Y	Z		PDM	Ctrl
ISE-INCL (PDM-Ctrl)									
ACC.L	N/A	198	3.659	9	–18	–33	Brainstem, CER4_5.L	0.571 ± 0.312	–1.095 ± 0.35
ACC.R	N/A	N/A	N/A	N/A	N/A	N/A	N/A	N/A	N/A
ACC	N/A	208	3.678	9	–18	–33	Brainstem, PHIPP.R, AMYG.R, HIPP.R	0.63 ± 0.309	–1.08 ± 0.356
ESE-ISE (PDM-Ctrl)									
ACC.L/ACC.R/ACC	N/A	N/A	N/A	N/A	N/A	N/A	N/A	N/A	N/A
ESE-INCL (PDM-Ctrl)									
ACC.L	PHIPP.L	770	4.292	–15	0	–29	INS.L, HIPP.L, PHIPP.R	–1.511 ± 0.251	–3.21 ± 0.292
	IFGtriang.R	556	3.897	39	33	18	MFG.R, PUT.R, CAU.R, ACC.R, INS.R	–2.653 ± 0.312	–4.382 ± 0.365
	THA.R	1076	3.94	6	–12	20	MCC.L, MFG.L, SFG.L, MCC.R, SFG.R, SMA.R, PreCG.L	–2.744 ± 0.348	–4.561 ± 0.344
ACC.R	N/A	579	4.484	–15	–15	–30	HIPP.L, PHIPP.L,	–1.119 ± 0.226	–2.652 ± 0.267
	IFGtriang.R	287	4.057	39	33	18	MFG.R, IFGoperc.R, INS.R, PreCG.R	–2.498 ± 0.323	–4.228 ± 0.34
	HIPP.R	241	3.839	30	–36	0	THA.R, THA.L, CAU.L	–2.727 ± 0.311	–4.262 ± 0.278
	SFG.R	261	3.497	18	24	42	MCC.L, MFG.L, SFG.L, SMA.R	–2.885 ± 0.399	–4.771 ± 0.349
ACC	N/A	652	4.478	–15	–15	–30	HIPP.L, PHIPP.L, PHIPP.R,	–1.14 ± 0.227	–2.672 ± 0.269
	MFG.R	489	4.21	42	33	21	IFGtriang.R, IFGoperc.R, INS.R, PreCG.R	–2.489 ± 0.313	–4.24 ± 0.355
	HIPP.R	391	3.934	30	–36	0	THA.R, THA.L, CAU.R, CAU.L	–2.707 ± 0.311	–4.305 ± 0.308

*ACC, anterior cingulate cortex; ISE, implicit social exclusion; INCL, inclusion; ESE, explicit social exclusion; PDM, primary dysmenorrhea; Ctrl, controls; PPI, psychophysiological interaction; ROI, region of interesting; MNI, Montreal Neurological Institute; AAL, automated anatomical labeling atlas; CER4_5, lobule IV, V of cerebellar hemisphere; PHIPP, parahippocampal gyrus; AMYG, amygdala; HIPP, hippocampal gyrus; INS, insula; IFGtriang, inferior frontal gyrus, triangular part; MFG, middle frontal gyrus; PUT, putamen; CAU, caudate nucleus; ACC, anterior cingulate cortex; THA, thalamus; MCC, middle cingulate cortex; SFG, superior frontal gyrus; SMA, supplementary motor area; PreCG, precentral gyrus; IFGoperc, inferior frontal gyrus, opercular part; SEM, standard error of mean; L/R, left/right hemisphere; N/A, non-applicable.*

We further evaluated the FC of the ACC-HIPP pathway using PPI analysis to reveal whether and how this specific pathway differs between PDM subjects and controls. We found that in the ISE-INCL contrast, PDM females showed significantly increased FC in the ACC.L-HIPP.L and ACC.R-HIPP.L circuits (Figure 3C). Furthermore, the combined ACC analysis, i.e., not separating left and right ACC, also showed significantly increased ACC-HIPP FC in the ISE-INCL contrast in PDM subjects (Figure 3C1). In the ESE-ISE contrast, overall ACC-HIPP FC did not differ significantly in PDM women (Figure 3D1), although FC of the ACC.L-HIPP.L and ACC.R-HIPP.L connections was significantly lower (Figure 3D). The results suggest that exclusion, whether explicit or implicit, triggered similar FC in the ACC-HIPP pathway in PDM and control females. In the ESE-INCL contrast, PDM subjects also showed significantly higher FC for all four ACC-HIPP connections (Figure 3E) as well as the combined analysis (Figure 3E1).

### Identical GM Volume in PDM and Control Subjects

Prolonged nociceptive input to the CNS has been shown to induce functional and structural alterations throughout the nervous system (Denk and McMahon, 2017). Having shown that significant differences occurred in the static and dynamic FC of PDM females, we investigated the GM volume of the ACC and HIPP using a voxel-based morphometry (VBM) approach. However, we found no significant differences between PDM subjects and controls (Figure 1D). Thus, the above abnormal connections between ACC and HIPP are not due to GM volume changes.

### Increased Writhing and Reduced Pain Threshold in a Pelvic Pain Rat Model

**FIGURE 4:**
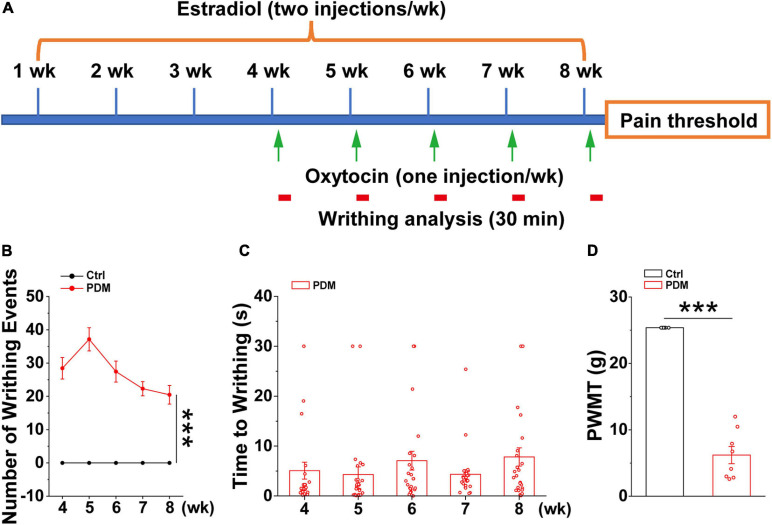
Behavioral evaluations in model rats. Schematic diagram showing the timeline and treatments used in generating the pelvic pain model, together with subsequent measurements. Starting from week four, oxytocin or saline was injected 24 h after estradiol benzoate for pelvic pain (PP) and control animals, respectively **(A)**. Writhing and pain threshold was measured 24 h after the last dose of oxytocin or saline, LFPs were recorded on days 58 and 59. **(B,C)** Number of writhing events (30 min time window) (4 weeks: PP: 28.442 ± 3.217; 5 weeks: 37.154 ± 3.485; 6 weeks: 27.442 ± 3.156; 7 weeks: 22.308 ± 2.133; 8 weeks: 20.481 ± 2.789) (*t* = 9.947, *p* = 1.627 × 10^– 18^) and latency (4 weeks: 5.072 ± 1.694 s; 5 weeks: 4.302 ± 1.544 s; 6 weeks: 7.069 ± 1.882 s; 7 weeks: 4.334 ± 0.950 s; 8 weeks: 7.820 ± 1.834 s) (*n* = 26). **(D)** Model rats show significantly reduced paw withdrawal mechanical threshold (PWMT) compared to control rats (*n* = 8–10) (PP: 6.199 ± 1.296 g; Control: 25.368 ± 0.000 g) (*t* = –16.667, *p* = 1.558 × 10^− 11^), suggesting increased pain sensitivity in model rats, which is in agreement with observations in PDM women. Values represent mean ± SEM. ***p < 0.001.

Given the ethical constraints of human studies, cellular and molecular studies using rodent models are beginning to be used to provide insights into the mechanisms that give rise to chronic pain (Zhuo, 2014). However, this has not been tested directly in PDM due to the lack of a chronic PDM rodent model. Therefore, we generated a chronic pelvic pain rat model that mimics dysmenorrhea-like pain experience based on an existing acute rodent PDM model (Chen et al., 2013; Jesuino et al., 2019), which was characterized by evaluating the levels of prostaglandin F2α and prostaglandin E2, endometrial thickness, and uterine artery blood flow velocity, etc., features that are similar to those found in human PDM (Yang et al., 2015). In this study, after 4 weeks of intraperitoneal (IP) estradiol benzoate injections, which promotes enhanced sensitivity of the uterus to oxytocin (Chen et al., 2013), oxytocin (PDM) or saline (Control) was IP-injected 24 h after estradiol benzoate and repeated for five consecutive weeks (Figure 4A). The rats given estradiol and oxytocin injections exhibited pain induced writhing, indicating abdominal/visceral pain (Supplementary Material 1), i.e., dysmenorrhea-like behavior, due to uterine contraction (Sun et al., 2002). The number of writhing events within a 30 min time window was evaluated (Figure 4B). Average writhing latency was less than 10 s after each oxytocin injection (Figure 4C). In agreement with findings suggesting that women with PDM have elevated pain reactivity (Iacovides et al., 2013), rat with pelvic pain showed a significantly decreased paw withdrawal mechanical threshold (PWMT) (Figure 4D), indicating that these rats are hypersensitive to pain. We next conducted electrophysiological and biochemical experiments in model and control rats to uncover molecular and cellular alterations induced by chronic pelvic pain.

### Alterations in Oscillatory Power in the ACC and HIPP

**FIGURE 5:**
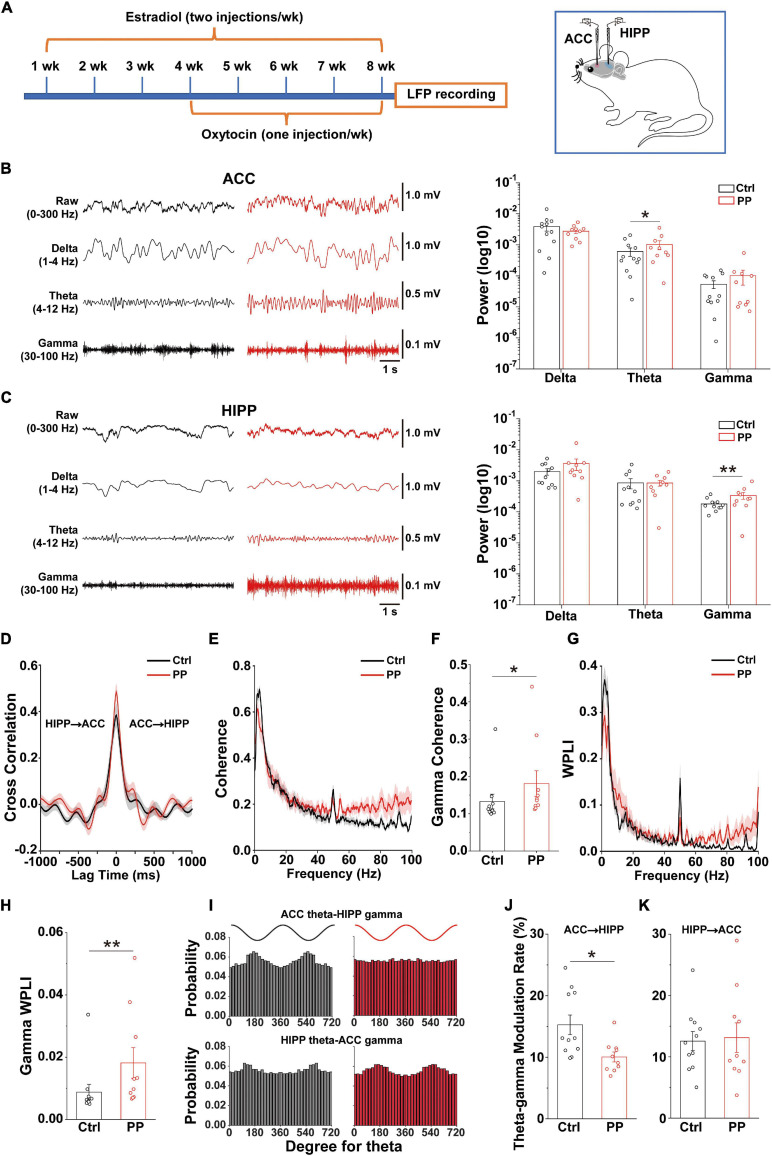
Altered oscillations and connectivity in ACC and HIPP of model rats. **(A)** Schematic diagram showing the timeline of model rat’s generation and dual channel *in vivo* LFP recordings in the left hemisphere. Starting from week 4, oxytocin was IP injected 24 h after injection of estradiol. The last dose of oxytocin was injected on day 57 and LFPs were recorded on days 58 and 59. **(B)** Representative traces of extracellular LFPs, as well as filtered delta, theta and gamma oscillations in the ACC of both groups (left). ACC oscillatory power in the theta band increases significantly in model compared to control rats, while delta and gamma oscillations remain identical between the two groups (*z* = –0.281, *p* = 0.779 for delta; *z* = –2.209, *p* = 0.027 for theta; *z* = 0.114, *p* = 0.909 for gamma; Mann–Whitney test). **(C)** Representative traces of extracellular LFPs, as well as filtered delta, theta and gamma oscillations in the HIPP of both groups (left). HIPP of model rats show significantly increased oscillatory power in the gamma band, while delta and theta oscillations remain identical between the two groups (*z* = –1.45, *p* = 0.147 for delta; *z* = –0.88, *p* = 0.379 for theta; *z* = –2.723, *p* = 0.006 for gamma; Mann–Whitney test). **(D)** The simultaneous LFP signals between ACC and HIPP have approximately symmetrical cross-correlation values at positive (ACC leading) and negative (HIPP leading) time lags in model and control animals, suggesting bidirectional communication between these two brain areas. **(E)** Averaged coherence curve between LFPs in ACC and HIPP. Notably, model rat differs significantly from control rats in the gamma band. **(F)** Gamma coherence between ACC and HIPP in model rats is significantly higher than that of controls (z = –2.289, *p* = 0.022; Mann–Whitney test). **(G)** Averaged WPLI curve between LFPs recorded in ACC and HIPP. **(H)** Gamma WPLI between ACC and HIPP is significantly higher in model rats (*z* = –2.711, *p* = 0.0067; Mann–Whitney test). **(I)** Probability distribution of cross-frequency theta-gamma coupling in both directions. **(J,K)** Quantification shows significantly reduced modulatory effect of ACC theta on HIPP gamma activity (*p* = 0.034), while modulation by HIPP theta of ACC gamma remains unchanged (*p* = 0.818; two-way ANOVA). Values represent mean ± SEM. *n* = 10–12; *p < 0.05, **p < 0.01.

Neuronal oscillatory activity, which is the neural basis of MRI and is preferentially sensitive to BOLD (Logothetis et al., 2001), is fundamental for the entrainment of precise temporal relationships between neuronal responses involved in cognition, perception and emotion (Mathalon and Sohal, 2015). To further evaluate whether the rat model is indeed representative of PDM, we examined whether abnormal neural oscillations occur in pelvic pain model rats by simultaneous dual-site LFP recording in the ACC and dorsal HIPP (Figure 5A). We found that the model rat demonstrated significantly enhanced theta power in the ACC (Figure 5B) and significantly enhanced gamma power in the HIPP (Figure 5C).

### Increased Gamma Coherence and WPLI Between ACC and HIPP in Model Rats

Functional coupling of oscillatory activities between pain processing and affective brain areas underlies the suffering associated with chronic pain (e.g., cognitional and emotional alterations), but this cannot easily be studied in PDM women. We thus examined electrical connectivity between the ACC and HIPP using cross-correlation analysis of LFPs (Engel et al., 2001). Model and control rats demonstrated similar correlation values in both ACC-leading and HIPP-leading directions (Figure 5D), suggesting the existence of similar bidirectional communication between the ACC and HIPP.

We next evaluated ACC-HIPP connectivity in the frequency domain (Figure 5E) by coherence analysis (Chen et al., 2021), and observed significantly increased coherence in the gamma range in model rats (Figure 5F). To better understand the connectivity in more precise phase ranges, we next used WPLI analysis (Vinck et al., 2011) which can reduce the contingency caused by the bidirectional connection. The result showed a significantly increased gamma-specific WPLI in model rats (Figures 5G,H), which was in agreement with the coherence analysis. Given that resting state FC indicated by BOLD output reflects the contributions of low frequency LFP signals and their dynamic changes (Shi et al., 2019), and that gamma band modulations co-localize with BOLD (Lachaux et al., 2007; Scheeringa et al., 2011), the enhanced synchronization observed between the ACC and HIPP in the gamma range reflects the increased FC shown by rs-fMRI in the ACC-HIPP pathway of PDM females.

### Reduced ACC Theta Modulation on HIPP Gamma Oscillations in Model Rats

The above results uncovered, in addition to increased ACC-HIPP connectivity, altered theta and gamma oscillations in the ACC and HIPP, respectively. The ACC is a key cortical region for pain perception (Vogt, 2005; Zhuo, 2008) and increased theta oscillation may indicate an alteration in how the ACC modulates HIPP activity. To confirm this, we next determined the strength of cross-frequency PAC between ipsilateral ACC and HIPP (Figure 5I). Despite an increase in the power of ACC theta oscillations, the modulating effect of ACC theta on HIPP gamma oscillations decreased significantly. At the same time, the modulatory effect by HIPP-theta on ACC-gamma oscillations remained unchanged (Figures 5J,K). Thus, chronic pelvic pain results in a reduction of ACC theta-HIPP gamma coupling, suggesting that pelvic pain, such as PDM may change top-down ACC informational input into the HIPP.

### Increased mEPSC Amplitude in ACC and HIPP in Model Rats

**FIGURE 6:**
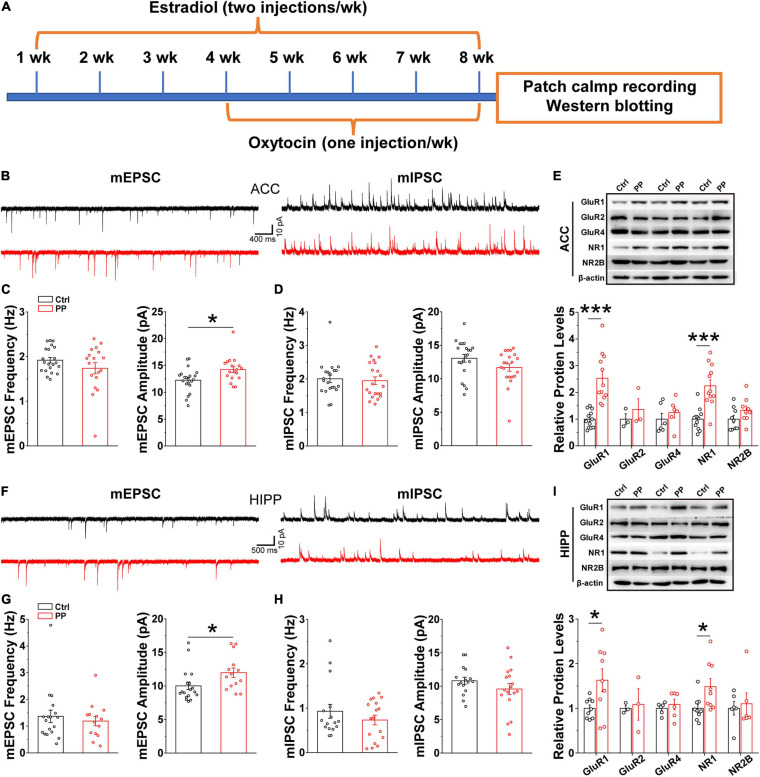
Increased mEPSC amplitude and levels of excitatory postsynaptic receptors in model rats. **(A)** Diagram showing model generation and patch clamp/western blot analysis. **(B)** Representative mEPSC (left) and mIPSC (right) traces recorded in the ACC. **(C)** Statistical analysis indicating significantly increased amplitude but not frequency of mEPSCs in model rat ACC (frequency: Control, 1.92 ± 0.06 Hz, PP, 1.71 ± 0.124 Hz, *p* = 0.136; amplitude: Control, 12.26 ± 0.48 pA, PP, 14.22 ± 0.55 pA, *p* = 0.012; Control, *n* = 21 cells of five rats; PP, *n* = 18 cells of five rats). **(D)** Identical mIPSC frequency and amplitude in control and model ACC (frequency: Control, 2.00 ± 0.11 Hz, PP, 1.95 ± 0.11 Hz, *p* = 0.20; amplitude: Control,13.06 ± 0.59 pA, PP, 11.67 ± 0.55 pA, *p* = 0.09; Control, *n* = 22 cells of five rats; PP, *n* = 22 cells of five rats). **(E)** Example of immunoblots of ACC extracts probed with anti-GluR1, GluR2, GluR4, NR1, and NR2B antibodies and quantification of the immunoblots revealing significant increases in levels of GluR1 (*p* = 0.014) and NR1 (*p* = 0.024), but not GluR2, GluR4 and NR2B. **(F)** Representative mEPSC (left) and mIPSC (right) traces in the HIPP. **(G)** Statistical analysis showing significantly increased mEPSC amplitude, but not frequency, in model rats (frequency: Control, 1.36 ± 0.12 Hz, *p* = 0.23; PP, 1.31 ± 0.15 Hz, *p* = 0.23; amplitude: Control, 10.89 ± 0.78 pA, PP, 12.67 ± 0.84 pA, *p* = 0.02; Control, *n* = 21 cells of five rats; PP, *n* = 20 cells of five rats). **(H)** Identical mIPSC frequency and amplitude in HIPP of control and model rats (frequency: Control, 0.98 ± 0.14, PP, 0.76 ± 0.18, *p* = 0.43; amplitude: Control, 10.71 ± 0.53, PP, 9.82 ± 0.59, *p* = 0.36; Control, *n* = 22 cells of 5 rats; PP, *n* = 22 cells of five rats). **(I)** Example of immunoblots probed with anti-GluR1, GluR2, GluR4, NR1, and NR2B antibodies and quantification of the immunoblots revealing a significant increase in levels of NR1 (*p* = 0.029). Values represent mean ± SEM. One-way ANOVA was used for mEPSCs and mIPSCs, and two-sample *t*-test for western blotting. *p < 0.05, ***p < 0.001.

Brain oscillations emerge as a consequence of local interactions between excitatory and inhibitory synaptic activities (Verret et al., 2012). Thus, the abnormal oscillations observed in the present study may implicate altered synaptic activities, because theta and gamma oscillatory activities relate to synaptic plasticity, in addition to network synchronization and memory formation (Tesche and Karhu, 2000; Buzsaki and Draguhn, 2004). We therefore examined whether or not abnormal excitatory and inhibitory synaptic activity in the ACC and HIPP underlie the aberrant oscillatory activity. Whole-cell patch-clamp recordings were conducted in brain slices containing either ACC or HIPP (Figure 6A). In voltage clamp mode and in the presence of TTX to block fast sodium channels and thus action potentials, the amplitude and frequency of mEPSCs and mIPSCs in the pyramidal neurons of the ACC and HIPP were measured. A significantly increased mEPSC amplitude was observed in pyramidal neurons in both the ACC and HIPP of model rats (Figures 6B,C,F,G), in agreement with the reported hyperactivity of ACC (Zhang et al., 2017; Zhou et al., 2018) and HIPP (Cardoso-Cruz et al., 2013) neurons during chronic pain. In contrast, mEPSC frequency in both ACC (Figure 6C) and HIPP (Figure 6G), as well as mIPSC amplitude and frequency (Figures 6D,H), was identical. These results suggest that increased excitation, which in turn leads to changes in the excitation/inhibition ratio, may contribute to abnormal oscillatory activities (Verret et al., 2012) in the ACC and HIPP.

### Upregulation in Levels of Excitatory Receptor Proteins in Model Rats

Postsynaptic excitatory responses are mediated mainly by two groups of glutamatergic receptors, AMPA and NMDA receptors (AMPARs, NMDARs) (Dai et al., 2019). The observed enhancement of mEPSC amplitude could be accounted for by increased levels of AMPARs and/or NMDARs. We next performed western blotting experiments with anti-AMPAR- and anti-NMDAR-subunit antibodies on ACC and HIPP lysates of model and control rats, which revealed significantly increased levels of both the AMPAR subunit, GluR1, and the NMDAR subunit, NR1, in the ACC (Figure 6E) and the HIPP (Figure 6I) of model rats. Together with the data showing increased mEPSC amplitude, these results suggest that upregulated levels of excitatory receptors may underlie hyperactivity of the ACC and HIPP in chronic PDM female.

## Discussion

We demonstrate here with two lines of evidence, human fMRI and animal electrophysiological, molecular and biochemical evaluations, that chronic pelvic pain, such as PDM, alters the FC of the ACC-HIPP pathway. In the first part of our study, in humans, we investigated the FC between the ACC and other brain areas, with an emphasis on the ACC-HIPP pathway, using fMRI in both rs-fMRI and T-fMRI, which can identify brain activation during social pain. In the second part of our study, we sought to fill a gap in the literature, i.e., the lack of a chronic animal model of PDM, and investigated the potential correlation between data obtained from female humans with PDM and the rat model that mimics PDM experience. We performed *in vivo* evaluation of LFPs in the ACC and HIPP, *in vitro* evaluation of whole-cell mEPSCs and mIPSCs, and tested levels of related proteins. Together, the results uncover changes caused by pelvic pain at the molecular, cellular, and systematic levels. The current findings represent, to the best of our knowledge, the first report linking alterations in the ACC-HIPP circuit in both human PDM subjects and a rodent model of pelvic pain. Therefore, this study provides an opportunity to determine common features that reliably contribute to pain perception and its modulation and emotional processing in pelvic pain, and should also allow testing of potential therapies for pelvic pain, including PDM, in the rat model.

Primary dysmenorrhea females show increased FC between the caudal ACC and primary somatosensory cortex, the perigenual ACC and caudate, and the subgenual ACC and medial prefrontal cortex (Liu et al., 2018). Our rs-fMRI results here extend this knowledge by revealing greater FC and FC variability between the ACC and HIPP in PDM females, which was further confirmed by LFP analysis that reveals increased communication in the theta and gamma range in the model rats. Specifically, the static and dynamic FC between the ACC and HIPP, structures critically involved in processing sensory, cognitive and affective components of pain (Rainville et al., 1997; Hutchison et al., 1999; Hashmi et al., 2013), were significantly enhanced in PDM females in rs-fMRI experiments, i.e., under basal conditions (without stimulation).

In T-fMRI experiments, we first revealed that PDM subjects have a lower, but controls a higher, level of CERCRU2 activation in the ESE condition compared to ISE. This result preliminarily suggests a role for the cerebellum in the response of implicit and explicit rejection, which requires confirmation in future studies due to the limitations of liberal correction used here. Moreover, our PPI analyses between the ACC and other brain regions demonstrate that PDM subjects have more ACC-brainstem/HIPP/THA/frontal lobe connections in the ISE-INCL and ESE-INCL. Furthermore, the PPI results suggest that ACC-HIPP coupling differs in a social experience-dependent manner in PDM women, representing higher ACC-HIPP connectivity overall in the ISE-INCL and ESE-INCL contrasts. Given that PPI values are negative here (Figure 3E), higher PPI values (FC) indicate that PDM females demonstrate a smaller reduction in connectivity during social exclusion, suggesting that PDM alters the response of the ACC-HIPP pathway under social pain conditions.

Interestingly, our previous behavioral evaluations showed that PDM females have a higher physical pain threshold in social exclusion situations (Yu et al., 2018). Therefore, the smaller reduction in FC between the ACC and the HIPP, as reported here, seems to be associated with a higher threshold of physical pain and reduced pain perception during social exclusion in PDM females. It is commonly accepted that negative emotional situations, e.g., social exclusion and pessimism, are associated with increased pain, while positive emotions are associated with decreased pain perception (Hanssen et al., 2013). In our study, social exclusion decreased pain perception in PDM females, which was consistent with findings in fibromyalgia patients (Canaipa et al., 2017). Social pain and physical pain have similar psychological and neurological processing (Eisenberger, 2012). The experience of long-term physical pain may lead to social-pain numbing (DeWall and Baumeister, 2006; Canaipa et al., 2016, 2017). Thus, social exclusion (social pain) has less influence on PDM females. Together, our previous and current studies implicate a potential link between ACC-HIPP connectivity and pain perception, highlighting the importance of evaluating pain networks, including those involving the ACC, in a broader social context (Sturgeon and Zautra, 2016), which, no doubt, will result in better treatment of pelvic pain.

It is noteworthy that we did not observe significantly altered GM volumes of either the ACC or HIPP in PDM females, which differs from a previous report (Tu et al., 2010), which showed increased GM volume in PDM females as measured by voxel-based morphometry in several brain areas, including the ACC and HIPP (right posterior), in the absence of pain. We think that this difference in findings might be due to the different pain history of the respective PDM cohorts, because the subjects used for GM volume estimation in our study have a PDM history of 5.29 ± 2.12 years, whereas there was a longer PDM history (10.19 ± 3.25 years) in the earlier study.

What is largely lacking in the field is a cellular and molecular understanding of how distinct areas of the brain interact to process sensory and affective components of pelvic pain. We found in the present study that ACC neurons exhibit hyperexcitation, in line with previous studies showing hyperactivity of the ACC in various physical and social pain conditions (Hutchison et al., 1999; Zhuo, 2014). Moreover, significantly increased theta power in the ACC of a model rat suggests that an increase in theta oscillations might be a common abnormality in both human (Lee et al., 2017; Ploner et al., 2017) and rodent models of pelvic pain. We also observed that ACC-HIPP synchrony of gamma oscillations increased significantly, which further suggested an upregulated FC of neuronal dynamics in this pathway, associated with reduced modulation by ACC theta oscillations of HIPP gamma oscillations.

Indeed, despite the stronger integration of the ACC-HIPP pathway in both human and model rat, we observed a smaller reduction in connectivity between ACC and HIPP (as shown by an increased FC value) during social exclusion in PDM women and reduced regulation of HIPP gamma by ACC theta in the PDM rat model. Considering ACC theta oscillations modulate HIPP high frequency activities via both direct and indirect ways in contextual processing during remote recall (Wirt and Hyman, 2019), it is likely that the increase in the ACC theta is an attempt by the regulatory circuit to compensate for the abnormally enhanced HIPP-gamma oscillations observed, although clearly this regulation is insufficient to restore normal levels. This might suggest that ineffective regulation of HIPP activity by the ACC may contribute, at least in part, to the abnormal ACC-HIPP FC and altered physical pain threshold in social exclusion situations (Yu et al., 2018) in PDM females. Thus, manipulating the ACC-HIPP circuit may ameliorate processing of physical and emotional pain in subjects with pelvic, such as PDM. Furthermore, our whole-cell patch clamp recording and western blotting analysis revealed an increased mEPSC amplitude associated with upregulated levels of NMDAR and AMPAR in both ACC and HIPP, thus highlighting some of the cellular and molecular mechanisms underlying pelvic pain.

There are differing reports on whether pain and social rejection are represented in the same (Eisenberger, 2015) or distinct (Woo et al., 2014) neural substrates; the present study suggests that physical and social pain may indeed interact in PDM subjects, leading to altered ACC-HIPP connectivity and physical- and social-pain processing. Although important questions remain open, human MRI together with LFP results obtained from model rat suggest a defective ACC-HIPP pathway in chronic pelvic pain. Therefore, the present study improves our understanding of how the coordination between ACC and HIPP becomes maladapted in chronic PDM, leading to aberrant processing of pain perception and pain-associated emotion. Thus, our work should also facilitate therapeutic targeting of pain-related psychiatric conditions.

## Data Availability Statement

The original contributions presented in the study are included in the article/Supplementary Material, further inquiries can be directed to the corresponding author/s.

## Ethics Statement

The studies involving human participants were reviewed and approved by Ethics Committee at South China Normal University. The patients/participants provided their written informed consent to participate in this study. The animal study was reviewed and approved by Ethics Committee at South China Normal University and Guangzhou University.

## Author Contributions

WY designed and performed the research, and wrote the manuscript. XW designed and performed the research, analyzed the data, and wrote the manuscript. YC performed the research, analyzed the data, and wrote the manuscript. ZL, AM, YS, and YP performed the research. JJ performed the research and analyzed the data. JC, BT, and MS performed the research and analyzed the data. CL contributed unpublished reagents and analytic tools. JS contributed to supervision and funding acquisition. LY contributed to guidance, funding acquisition, and wrote the manuscript. All the authors contributed to the article and approved the submitted version.

## Conflict of Interest

The authors declare that the research was conducted in the absence of any commercial or financial relationships that could be construed as a potential conflict of interest.

## Footnotes

1 https://www.fil.ion.ucl.ac.uk/spm/software/spm12/

2 http://www.rfmri.org/
